# Effects of low skeletal muscle mass and sarcopenic obesity on albuminuria: a 7-year longitudinal study

**DOI:** 10.1038/s41598-020-62841-y

**Published:** 2020-04-01

**Authors:** Jee Hee Yoo, Gyuri Kim, Sung Woon Park, Min Sun Choi, Jiyeon Ahn, Sang-Man Jin, Kyu Yeon Hur, Moon-Kyu Lee, Mira Kang, Jae Hyeon Kim

**Affiliations:** 1 Division of Endocrinology and Metabolism, Department of Medicine, Samsung Medical Center, Sungkyunkwan University School of Medicine, Seoul, Republic of Korea; 2 Department of Internal Medicine, Yonsei University Wonju College of Medicine, 20 Ilsan-ro, Wonju, 26426 Republic of Korea; 3 Department of Digital Health, SAIHST, Sungkyunkwan University, Seoul, 06351 Republic of Korea; 4 Center for Health Promotion, Samsung Medical Center, Sungkyunkwan University School of Medicine, Seoul, Republic of Korea

**Keywords:** Cardiology, Endocrinology, Nephrology, Risk factors

## Abstract

We aimed to identify the association between low skeletal muscle, sarcopenic obesity, and the incidence of albuminuria in the general population using a longitudinal study. Data from 29,942 subjects who underwent two or more routine health examinations from 2006 to 2013 were retrospectively reviewed. Relative skeletal muscle mass was presented using the skeletal muscle mass index (SMI), a measure of body weight-adjusted appendicular skeletal muscle mass estimated by bioelectrical impedance analysis. The cumulative incidence of albuminuria was 981 (3.3%) during the 7-year follow-up period. The hazard ratio of incident albuminuria was 1.44 (95% CI: 1.22–1.71, *p* for trend <0.001) in the lowest SMI tertile relative to the highest SMI tertile after multivariable adjustment. After additionally adjusting for general and central obesity, the hazard ratio was 1.35 (95% CI: 1.13–1.61, *p* for trend = 0.001) and 1.30 (95% CI: 1.08–1.56, *p* for trend = 0.003), respectively. Furthermore, the risk of developing albuminuria was much higher in the sarcopenic obesity group (HR: 1.49, 95% CI: 1.21–1.81, *p* for trend <0.001) compared to the other groups. Sarcopenic obesity, as well as low skeletal muscle, may lead to albuminuria in general populations.

## Introduction

Low muscle mass or sarcopenia is defined as a progressive decrease in muscle mass and strength by aging, which can lead to the progression of chronic metabolic diseases and eventually leads to morbidity and mortality^1^. Recently, sarcopenia and obesity have been reported to synergistically worsen functional decline and outcomes than either condition alone. Moreover, the population is getting older and the prevalence of obesity is rapidly increasing in Asia^2,3^. Therefore, sarcopenia and obesity have emerged as a major health issue worldwide, including Korea^4,5^.

Albuminuria is a risk marker for endothelial cell dysfunction, which leads to cardiovascular and kidney disease not only in patients with diabetes or hypertension but also in the general population^6–10^. Moreover, albuminuria is independently associated with all-cause mortality^11,12^.

Several cross-sectional studies suggest that both sarcopenia and obesity are individually associated with the prevalence of albuminuria in subjects with diabetes and hypertension^13,14^. Even in healthy populations, both sarcopenia and obesity have consistently emerged as significant risk factors for albuminuria in cross-sectional settings^15–19^.

However, the causal relationship between low muscle mass and albuminuria in longitudinal data has not been elucidated. Even though obesity may exaggerate functional decline with sarcopenia, the association between sarcopenic obesity and albuminuria has not been established.

Therefore, this study examines whether subjects with low skeletal muscle mass have higher incidences of albuminuria in a large population-based 7-year longitudinal study. In addition, we further explored the combined effects of sarcopenia and obesity on the risk of developing albuminuria.

## Results

### Baseline characteristics of study subjects according to sex-specific SMI tertiles

First, we investigated the sequential association of skeletal muscle mass using SMI tertiles and incidence of albuminuria, regardless of obesity. The mean follow-up duration was 29.3 ± 19.0 months (range, 2.0 years to 7.0 years). The study population comprised 29,942 subjects with a mean age of 49.9 ± 7.9 years and mean BMI of 23.6 ± 2.9 kg/m^2^; 54.0% were male. The characteristics of the study participants according to the sex-specific SMI tertiles are summarized in Table 1. Subjects in the lowest SMI tertile group tended to be older, more obese, and have worse lipid parameters compared to those in the highest SMI tertile group. Blood pressure, HbA1c, FPG, CRP, and HOMA-IR levels showed increasing trends as SMI tertiles decreased.

**Table 1:** Baseline characteristics according to sex-specific SMI tertiles.

	SMI tertiles			P value
	Highest (n = 9,980)	Middle (n = 9,982)	Lowest (n = 9,980)	
Age, years (SD)	48.0 (7.3)	49.9 (7.3)	52.1 (8.5)	<0.001
Sex				1.000
Men, n (%)	5387 (54.0)	5388 (54.0)	5387 (54.0)	
Women, n (%)	4593 (46.0)	4594 (46.0)	4593 (46.0)	
Skeletal muscle mass index (SD)	33.4 (2.4)	30.6 (2.2)	28.1 (2.6)	<0.001
Men	35.2 (1.3)	32.6 (0.5)	30.2 (1.2)	
Women	31.2 (1.4)	28.3 (0.6)	25.6 (1.3)	
Body weight, kg (SD)	61.6 (10.2)	64.8 (10.8)	68.3 (12.1)	<0.001
BMI, kg/m^2^ (SD)	21.7 (2.2)	23.5 (2.2)	25.6 (2.8)	<0.001
Waist circumference, cm (SD)	78.0 (7.7)	82.3 (8.0)	87.2 (8.8)	<0.001
Current smoker, n (%)	1942 (23.6)	1801 (22.5)	1745 (22.0)	0.004
Regular exercise, n (%)	1939 (20.5)	1616 (17.2)	1523 (16.3)	<0.001
SBP, mmHg (SD)	111.3 (14.7)	116.9 (15.6)	121.0 (15.7)	<0.001
DBP, mmHg (SD)	71.1 (11.0)	73.2 (11.1)	75.0 (10.9)	<0.001
Total cholesterol, mg/dL (SD)	190.3 (32.0)	197.3 (32.8)	203.1 (35.5)	<0.001
HDL-C, mg/dL (SD)	59.7 (15.3)	55.5 (14.1)	53.2 (13.4)	<0.001
Triglycerides, mg/dL (SD)	102.5 (60.1)	124.8 (76.6)	141.9 (82.4)	<0.001
LDL-C, mg/dL (SD)	116.7 (28.3)	124.5 (28.8)	129.8 (30.8)	<0.001
Fasting glucose, mg/dL (SD)	91.4 (14.8)	93.9 (15.6)	97.6 (18.8)	<0.001
HbA1c, % (SD)	5.4 (0.6)	5.5 (0.6)	5.6 (0.7)	<0.001
CRP, mg/L (SD)	0.10 (0.36)	0.11 (0.27)	0.14 (0.33)	<0.001
Insulin, uIU/mL (SD)	6.8 (3.5)	8.1 (3.7)	9.8 (5.3)	<0.001
C-peptide, ng/mL (SD)	1.44 (0.57)	1.73 (0.68)	2.06 (0.83)	<0.001
HOMA-IR (SD)	1.54 (1.04)	1.88 (0.98)	2.39 (1.49)	<0.001
BUN, mg/dL (SD)	13.3 (3.4)	13.4 (3.4)	13.7 (3.4)	<0.001
Creatinine, mg/dL (SD)	0.88 (0.17)	0.87 (0.17)	0.86 (0.16)	<0.001
eGFR, mL/min per 1.73 m^2^ (SD)	83.8 (12.9)	84.0 (13.0)	84.5 (13.6)	0.001
Urinary albumin-to-creatinine ratio, mg/g (SD)	6.0 (5.1)	6.5 (5.4)	7.3 (5.9)	<0.001

BMI body mass index, BUN blood urea nitrogen, CRP c-reactive protein, DBP diastolic blood pressure, eGFR estimated glomerular filtration rate, HDL-C high-density lipoprotein cholesterol, HOMA-IR homeostasis model assessment of insulin resistance, LDL-C low-density lipoprotein cholesterol, SBP systolic blood pressure, SD standard deviation, SMI skeletal muscle mass index.

### Association between baseline SMI and incident albuminuria

**Figure 1:**
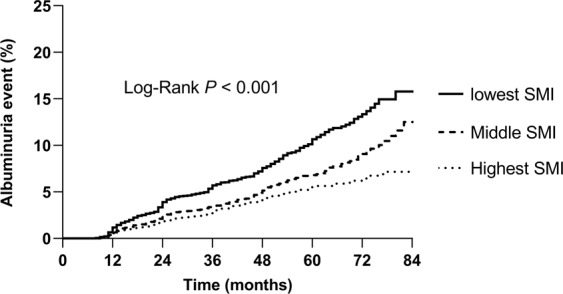
Albuminuria incidence according to sex-specific SMI tertiles (Kaplan-Meier analysis). *SMI* skeletal muscle mass index.

**Figure 2:**
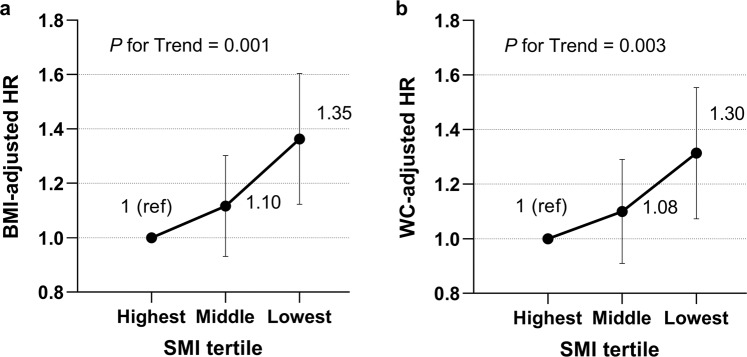
Adjusted HRs for incident albuminuria according to sex-specific SMI tertiles. Adjustment variables for multivariable-adjusted HRs included main covariates (age, sex, SBP, HbA1c, LDL-C, HOMA-IR, CRP, eGFR and use of antihypertensive medication) and obesity categorized by (**a**) BMI (≥27.5) or (**b**) waist circumference (WC ≥ 90 cm for men, ≥85 cm for women). *ACEi* angiotensin converting enzyme inhibitor, *ARB* angiotensin receptor blocker, *BMI* body mass index, *CRP* c-reactive protein, *eGFR* estimated glomerular filtration rate, *HOMA-IR* homeostasis model assessment of insulin resistance, *HRs* hazard ratios, *LDL-C* low-density lipoprotein cholesterol, *SMI* skeletal muscle mass index, *WC* waist circumferences.

Among the 29,942 subjects, 981 (3.3%) developed albuminuria during the 7-year follow-up period. The cumulative incidence of albuminuria significantly increased in subjects with lower tertiles of baseline SMI compared with those with the highest tertile (Fig. 1, p < 0.001 by log-rank test). To investigate independent association between the baseline SMI and incidence of albuminuria, cox proportional hazard regression analyses were performed (Table 2). In comparison with the highest SMI tertile, the lowest SMI tertile was independently associated with the incidence of albuminuria with a hazard ratio (HR) of 1.44 [95% confidence interval (CI), 1.22–1.71)] after adjustment for age, sex, SBP, HbA1c, LDL-C, HOMA-IR, CRP, eGFR and use of antihypertensive medications (model 4). The associations remained significant after further adjustment for general or central obesity. The hazard ratios of having albuminuria were 1.35 (95% CI: 1.13–1.61, *p* for trend = 0.001, Fig. 2a) and 1.30 (95% CI: 1.08–1.56, *p* for trend = 0.003, Fig. 2b) in the lowest SMI tertile compared with the highest SMI tertile after adjusting for obesity defined by BMI or WC, respectively.

**Table 2:** Association between sex-specific SMI tertiles and albuminuria incidence.

	Model 1	Model 2	Model 3	Model 4
Highest SMI	1 (ref)	1 (ref)	1 (ref)	1 (ref)
Middle SMI	1.24 (1.05–1.48)	1.20 (1.01–1.43)	1.15 (0.96–1.37)	1.12 (0.93–1.33)
Lowest SMI	1.97 (1.68–2.30)	1.81 (1.54–2.13)	1.56 (1.32–1.84)	1.44 (1.22–1.71)
*P* for Trend	<0.001	<0.001	<0.001	<0.001

Model 1: crude.

Model 2: adjusted for age and sex.

Model 3: adjusted for Model 2 + SBP, HbA1c, LDL-C, HOMA-IR, CRP, and eGFR.

Model 4: adjusted for Model 3 + use of antihypertensive medication.

*CRP* c-reactive protein, *eGFR* estimated glomerular filtration rate, *HOMA-IR* homeostasis model assessment of insulin resistance, *LDL-C* low-density lipoprotein cholesterol, *SMI* skeletal muscle mass index.

**Figure 3:**
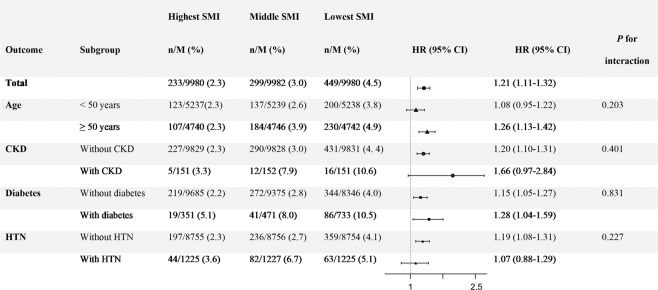
Subgroup analyses of association between sex-specific SMI tertiles and albuminuria incidence (Subgroups were analyzed for model 4). *CKD* chronic kidney disease, *HTN* hypertension, *HR* hazard ratio, SMI skeletal muscle mass index.

In the subgroup analyses, the strong inverse association between SMI tertiles and incident albuminuria remained regardless of age over 50 years old, presence of diabetes, presence of hypertension or presence of CKD (*p* for interaction >0.05, model 4, Fig. 3).

### Association between sarcopenic obesity group and incident albuminuria

Next, we investigated the incidence of albuminuria with sarcopenic obesity to support the combined exaggerated risk of sarcopenia on obesity.

Baseline characteristics stratified by body composition (according to WC and SMI) are shown in Table S1. Ten point three percentages of subjects had sarcopenia but not obesity, 14.5% of subjects had obesity but not sarcopenia, and 9.2% had both (sarcopenic obesity). Sarcopenia in the absence of obesity was associated with an increased risk of albuminuria (Table 3, adjusted HR: 1.35 [95% CI, 1.09–1.67]) after adjustment for age, sex, SBP, HbA1c, LDL-C, HOMA-IR, CRP, eGFR and use of antihypertensive medications (model 4). Obesity in the absence of sarcopenia was also associated with an increased risk of albuminuria (adjusted HR: 1.38 [95% CI, 1.15–1.65], model 4). Furthermore, sarcopenic obesity was associated with the highest risk of albuminuria compared with the other three categories of body composition (adjusted HR: 1.49 [95% CI, 1.21–1.81], model 4). Table 4 also shows the similar patterns of association with sarcopenic obesity defined by BMI and SMI and the risk of albuminuria. Compared with the non-sarcopenia/non-obese (optimal) group, the sarcopenic obesity group had the highest risk for albuminuria (adjusted HR: 1.53 [95% CI, 1.23–1.91]), followed by the group with obesity in the absence of sarcopenia (adjusted HR: 1.40 [95% CI, 1.04–1.88]), and the group with sarcopenia in the absence of obesity (adjusted HR: 1.23 [95% CI, 1.03–1.46]) in fully adjusted model (model 4).

**Table 3:** Association between sarcopenic obese status (according to WC and SMI) and albuminuria incidence.

	Model 1	Model 2	Model 3	Model 4
Optimal	1 (ref)	1 (ref)	1 (ref)	1 (ref)
Sarcopenic	1.59 (1.30–1.94)	1.50 (1.22–1.86)	1.40 (1.13–1.74)	1.35 (1.09–1.67)
Obese	1.65 (1.39–1.96)	1.59 (1.33–1.89)	1.44 (1.20–1.72)	1.38 (1.15–1.65)
Sarcopenic obese	2.29 (1.91–2.76)	2.09 (1.73–2.52)	1.63 (1.34–1.98)	1.49 (1.21–1.81)
*P* for Trend	<0.001	<0.001	<0.001	<0.001

Model 1: crude.

Model 2: adjusted for age and sex.

Model 3: adjusted for Model 2 + SBP, HbA1c, LDL-C, HOMA-IR, CRP, and eGFR.

Model 4: adjusted for Model 3 + use of antihypertensive medication.

*CRP* c-reactive protein, *eGFR* estimated glomerular filtration rate, *HOMA-IR* homeostasis model assessment of insulin resistance, *LDL-C* low-density lipoprotein cholesterol*, SMI* skeletal muscle mass index, *WC* waist circumference.

**Table 4:** Association between sarcopenic obese status (according to BMI and SMI) and albuminuria incidence.

	Model 1	Model 2	Model 3	Model 4
Optimal	1 (ref)	1 (ref)	1 (ref)	1 (ref)
Sarcopenic	1.51 (1.29–1.78)	1.42 (1.19–1.69)	1.28 (1.07––1.53)	1.23 (1.03–1.46)
Obese	1.75 (1.32–2.33)	1.76 (1.31–2.34)	1.47 (1.10–1.97)	1.40 (1.04–1.88)
Sarcopenic obese	2.36 (1.91–2.93)	2.27 (1.84–2.81)	1.69 (1.35–2.09)	1.53 (1.23–1.91)
*p* for Trend	<0.001	<0.001	<0.001	<0.001

Model 1: crude.

Model 2: adjusted for age and sex.

Model 3: adjusted for Model 2 + SBP, HbA1c, LDL-C, HOMA-IR, CRP, and eGFR.

Model 4: adjusted for Model 3 + use of antihypertensive medication.

*BMI* body mass index, *CRP* c-reactive protein, *eGFR* estimated glomerular filtration rate, *HOMA-IR* homeostasis model assessment of insulin resistance, *LDL-C* low-density lipoprotein cholesterol, *SMI* skeletal muscle mass index.

## Discussion

In this large study of 29,942 Korean adults, we found that subjects with a low SMI were associated with a 30% to 35% increased risk of developing albuminuria after adjusting for potential confounders, including obesity. The relationship between SMI and incident albuminuria significantly remained in various subgroups (i.e., age, diabetes, CKD and hypertension). Furthermore, subjects with sarcopenia combined with obesity had a higher risk of developing albuminuria than subjects with obesity or sarcopenia alone.

Previous studies have shown the association between sarcopenia, obesity, and albuminuria in a healthy population^16,17,20^. Han *et al*.^16^ analyzed 2,326 subjects and showed that sarcopenia increased odds ratio of 1.6 for albuminuria, even after adjusting for multiple confounding factors. Also, the sarcopenic obesity group had a significantly higher odds ratio compared to other groups. Another study analyzed 2,158 subjects and supported the results that low SMI was associated with a 2.9-fold odds ratio for albuminuria after adjusting multiple confounding factors^17^. However, the studies were cross-sectional in design and limited by small samples sizes.

Until now, only a single longitudinal study has investigated the association between SMI and the incidence of albuminuria^20^. In agreement with our study, sarcopenia was significantly associated with incidence of albuminuria. However, they used a semi-quantitative urine dipstick test, instead of a quantitative test, as was employed in our study. We investigated the development of albuminuria defined as urine ACR > 30 mg/g. The accuracy of dipsticks in diagnosing microalbuminuria is much lower than using the albumin concentration method^21^. In the present study, we additionally analyzed the risk of albuminuria by sarcopenic obesity status to evaluate the risk of sarcopenia in obese subjects. Thus, to the best of our knowledge, this is the first report investigating the relationship between sarcopenic obesity as well as relative skeletal muscle mass determined by SMI, and albuminuria development using a large, general population-based 7-year longitudinal dataset.

Low SMI as a risk factor for albuminuria has not been fully evaluated. However, insulin resistance and endothelial dysfunction due to loss of muscle mass have been established as potential mechanisms behind albuminuria in both non-diabetic^22–27^ and diabetic subjects^28^. First, skeletal muscle mass is the largest insulin-sensitive tissue in the body^29^. Thus, loss of muscle mass and strength lead to exacerbated insulin resistance, which can increase profibrotic elements and vascular growth factors involved in damaging glomerular function and eventually end in albumin leakage^25,30^. Second, low skeletal muscle mass is associated with decreased adipocytokines and increased inflammation, which can induce endothelial senescence and dysfunction^27^. This dysregulation in endothelial cells can damage the glomerulus and increase the permeability of albumin^26^.

Obesity is known to increase albuminuria by triggering cascades of events including increased inflammatory markers, reactive oxygen species, and insulin resistance, similar to sarcopenia^31,32^. Moreover, the mechanism leads to the development of sarcopenia. Sarcopenia, in turn, is associated with physical inactivity, which leads to an increase in obesity^33^. Either sarcopenia or obesity could be the initial step in the development of sarcopenic obesity, creating a vicious cycle, which together can lead to widespread organ damage and conditions such as albuminuria^34^.

A key strength of this study is the large sample size of 29,942 subjects, which represents a valuable dataset that can provide more reliable results compared to smaller studies. Moreover, our study provides strong evidence of a relationship between skeletal muscle mass and albuminuria by adjusting for variable confounding factors and conducting stratification analyses. We defined albuminuria quantitatively, as having an ACR of more than 30 mg/g, which could reflect initiation of microalbuminuria. Finally, we investigated not only the association of sarcopenia with albuminuria, but also the effects of continuous value of relative skeletal muscle mass determined by SMI, combined effect of sarcopenia and obesity on developing albuminuria.

Our study is limited by the lack of repeated measurements of ACR, thus transient albuminuria could not be excluded^35^. Second, the CKD patients were not excluded. However, whether CKD was present or not, the SMI tertiles and incidence of albuminuria were significantly associated in the subgroup analysis. Third, the subjects in this study were all Korean individuals who participated in routine health evaluations; therefore, the results may not be generalizable to other settings or other ethnicities. Also, due to the lack of information of the type of antihypertensive medication, we showed analyses after adjustment of the use of antihypertensive medication rather than specific use of angiotensin-converting enzyme inhibitor (ACEi)s or angiotensin receptor blocker (ARB)s, which also can affect the outcome of the study.

In conclusion, our analyses support that low skeletal muscle mass could act as a prognostic indicator for albuminuria. Also, sarcopenia and obesity combined together increase the risk of developing albuminuria compared to subjects with sarcopenia or obesity alone.

## Methods

### Study populations

The study population is consisted of participants who underwent two or more routine health evaluations at the Samsung Medical Center (SMC, Seoul, Republic of Korea) from August 2006 to August 2013.

Initially, 60,843 subjects were identified. Subjects with missing data for baseline skeletal muscle mass (n = 2,674) or laboratory data including serum creatinine and urine albumin-to-creatinine ratio (ACR, n = 26,283), and subjects with ACR more than 30 mg/g (n = 1820) at baseline were excluded. After excluding ineligible participants, 29,942 subjects were included in the final study population (Fig. S1). The Institutional Review Board (IRB) of SMC approved this study protocol (No. 2018-02-143) and the informed consent requirement was waived by the IRB, because the study information was de-identified. The protocol for the study was in accordance with the guidelines of the Helsinki Declaration.

### Measurement of clinical variables and biochemical data

Each subject completed a self-administered questionnaire that covered their prior medical history, surgical history, prescribed medications, smoking status, and exercise history. Smoking status was categorized as never, past smoker, or current smoker. Exercise status was assessed as none or regular exercise (≥3 days/week).

Subjects underwent anthropometric evaluation including weight and height with light clothing. Waist circumference (WC) was measured at the narrowest level between the upper iliac crest and lowest rib after normal expiration. Body mass index (BMI, kg/m^2^) was calculated, and systolic (SBP) and diastolic blood pressures (DBP) were measured in a sitting position using a sphygmomanometer after a 5-minute rest period and expressed as the mean of two readings^36^.

Blood samples were collected after a 12-hour overnight fast. Detailed methods regarding measurements of blood laboratory profiles were performed as described in the previous study^37^. Homeostasis model assessment of insulin resistance (HOMA-IR) was calculated by the following formula: fasting plasma insulin (μIU/mL) × fasting plasma glucose (FPG, mg/dL)/405^38^. ACR was measured in a spot urine collection, and the ratio (mg/g) was used for the assessment of clinical stages of albuminuria (normoalbuminuria, ACR < 30; albuminuria, ACR ≥ 30)^39^.

Diabetes was defined as having FPG ≥ 126 mg/dL or HbA1c ≥ 6.5% or using diabetes medication^40^. Hypertension was defined as having blood pressure ≥140/90 mmHg or taking antihypertensive medication^41^.

### Measurement of skeletal muscle mass index

After an overnight fast, bioelectrical impedance analysis (BIA) was conducted to determine appendicular skeletal muscle mass (ASM) for each limb (kg) using a multifrequency BIA device according to the manufacturer’s instructions (InBody 720; Biospace Inc., Seoul, Korea)^36,42,43^.

According to a modified formula in the previous study, the skeletal muscle mass index (SMI) was calculated by dividing the sum of the ASM in the bilateral upper and lower four limbs (kg) by body weight (kg) and expressed as a percentage (=total ASM/body weight × 100%)^44^. Subjects were divided into three groups based on sex-specific SMI tertiles: lowest (22.2–31.7), middle (31.8–33.7), and highest (33.8–42.6) for men and lowest (19.0–27.4), middle (27.5–29.6), and highest (29.7–40.2) for women.

### Definition of sarcopenia and obesity

Sarcopenia was defined as <1 SD (standard deviation) below the mean of the sex-specific SMI for a young reference group (aged 18–40)^1^. The cutoff point for sarcopenia was 30.5% in men and 27.0% in women. General obesity was defined using BMI ≥ 27.5 and central obesity was defined using WC of ≥90 cm for men and ≥85 cm for women^45,46^.

A composite of 4 mutually exclusive categories of body composition (sarcopenic obese status, obese status by WC) was generated. These were (1) optimal body composition (i.e., non-obese and non-sarcopenic), (2) sarcopenic (ie, non-obese), (3) obese (ie, non-sarcopenic), and (4) sarcopenic obesity.

### Statistical analysis

Data are expressed as the mean ± SD for continuous variables and as a percentage for categorical variables^47^. Comparisons between baseline characteristics according to sex-specific SMI tertiles and sarcopenic obese status were made using a one-way analysis of variance (ANOVA) with Bonferroni’s method for continuous variables, and chi-square test with linear by linear analysis was used in categorical variables^47^. Cumulative event rates for incident albuminuria were estimated by Kaplan-Meier survival curves, and equalities were compared with the log-rank test. Cox proportional hazard analysis was performed to determine independent associations between baseline sex-specific SMI and the development of albuminuria^36^. For multivariable-adjusted analyses, model 1 was non-adjusted, model 2 was adjusted for age and sex, and model 3 was further adjusted for HbA1c, SBP, low density lipoprotein cholesterol (LDL-C), HOMA-IR, c-reactive protein (CRP), and eGFR. Model 4 was further adjusted for the use of antihypertensive medication. Furthermore, obesity defined by BMI and WC was also adjusted for multivariable cox regression analysis. In addition, we performed subgroup analyses defined by age (<50 years, 50≥ years) or presence of CKD, diabetes, or hypertension. Cox proportional hazard analysis was again performed to evaluate the risk for incidence of albuminuria according to the sarcopenic obese status after adjusting for confounding variables. All statistical analyses were performed using IBM SPSS version 26.0 for windows (SPSS Inc, Chicago, IL).

## Supplementary information

Supplementary Information.

## Data Availability

The datasets used and/or analyzed during the current study are available from the corresponding author on reasonable request.
