# Supplementary material for: Effects of low skeletal muscle mass and sarcopenic obesity on albuminuria: a 7-year longitudinal study
Source: Sci Rep. 2020 Apr 1;10:5774. doi: 10.1038/s41598-020-62841-y (PMC7113302; doi:10.1038/s41598-020-62841-y)
Supplement: Supplementary file 1 — Supplementary Information. [file 41598_2020_62841_MOESM1_ESM.pdf]

Supplementary information

Effects of low skeletal muscle mass and sarcopenic obesity on albuminuria: a 7-year longitudinal study

Jee Hee Yoo[1,2*], Gyuri Kim[1*], Sung Woon Park[1], Min Sun Choi[1], Jiyeon Ahn[1], Sang-Man Jin[1], Kyu Yeon Hur[1], Moon-Kyu Lee[1], Mira Kang[3,4], Jae Hyeon Kim[1]

[1]Division of Endocrinology and Metabolism, Department of Medicine, Samsung Medical Center, Sungkyunkwan University School of Medicine, Seoul, Republic of Korea

[2]Department of Internal Medicine, Yonsei University Wonju College of Medicine, 20 Ilsan-ro, Wonju, 26426, Republic of Korea

[3]Department of Digital Health, SAIHST, Sungkyunkwan University, Seoul 06351, Republic of Korea

[4]Center for Health Promotion, Samsung Medical Center, Sungkyunkwan University School of Medicine, Seoul, Republic of Korea

Correspondence

Jae Hyeon Kim

Division of Endocrinology and Metabolism,

Department of Medicine, Samsung Medical Center,

Sungkyunkwan University School of Medicine

81, Irwon-ro, Gangnam-gu, Seoul 06351, Republic of Korea

Email: jaehyeonkim26@gmail.com

Phone: 82-2-3410-1580

Mira Kang

Department of Health Promotion Center,

Samsung Medical Center,

Sungkyunkwan University School of Medicine,

81, Irwon-ro, Gangnam-gu, Seoul 06351, Republic of Korea

Email: mira90.kang@samsung.com

Table S1. Baseline characteristics according to sarcopenic obese status (according to WC and SMI)

	Body composition				P value
	Optimal (n = 17,382)	Sarcopenic (n = 3,095)	Obese (n = 4,335)	Sarcopenic obese (n = 2,740)	
**Age, years (SD)**	49.3 (7.5)	53.2 (8.6)	48.5 (7.2)	50.6 (8.7)	< 0.001
**Sex**					< 0.001
**Men, n (%)**	9260 (61.2)	511 (3.4)	3840 (25.4)	1514 (10.0)	
**Women, n (%)**	8122 (65.4)	2584 (20.8)	495 (4.0)	1226 (9.9)	
**Skeletal muscle mass index (SD)**	31.9 (2.6)	26.6 (1.9)	31.4 (1.2)	27.2 (2.5)	< 0.001
**Men**	33.5 (1.8)	29.3 (1.1)	31.6 (0.9)	28.8 (1.1)	
**Women**	29.7 (1.8)	25.8 (1.1)	27.9 (0.6)	24.3 (1.5)	
**Body weight, kg (SD)**	63.7 (10.3)	60.8 (8.4)	85.3 (6.9)	80.8 (10.8)	< 0.001
**BMI, kg/m^2 (SD)**	22.7 (2.4)	24.1 (2.0)	28.6 (0.9)	29.6 (2.1)	< 0.001
**Waist circumference, cm (SD)**	80.8 (8.1)	82.4 (7.4)	95.5 (4.8)	96.3 (7.4)	< 0.001
**Current smoker, n (%)**	3117 (22.0)	159 (5.9)	1338 (41.2)	528 (24.9)	< 0.001
**Regular exercise, n (%)**	3170 (19.2)	526 (18.2)	583 (14.2)	416 (16.5)	< 0.001
**SBP, mmHg (SD)**	115.6 (15.3)	119.3 (16.2)	123.3 (14.3)	125.2 (15.5)	< 0.001
**DBP, mmHg (SD)**	72.6 (11.1)	72.8 (10.9)	78.1 (10.5)	77.7 (10.6)	< 0.001
**Total cholesterol, mg/dL (SD)**	194.6 (32.9)	204.5 (35.4)	197.4 (34.4)	203.3 (36.7)	< 0.001
**HDL-C, mg/dL (SD)**	57.0 (14.7)	57.0 (13.9)	46.5 (10.5)	49.4 (11.7)	< 0.001

**Triglycerides, mg/dL (SD)**	116.9 (71.5)	125.6 (69.5)	172.9 (107.7)	162.4 (90.3)	< 0.001
**LDL-C, mg/dL (SD)**	121.6 (29.1)	129.5 (31.3)	127.2 (29.0)	130.8 (31.3)	< 0.001
**Fasting glucose, mg/dL (SD)**	93.2 (15.7)	95.5 (17.8)	100.1 (18.4)	101.9 (21.0)	< 0.001
**HbA1c, % (SD)**	5.4 (0.6)	5.6 (0.6)	5.7 (0.7)	5.8 (0.8)	< 0.001
**CRP, mg/L (SD)**	0.11 (0.31)	0.14 (0.40)	0.14 (0.23)	0.18 (0.29)	< 0.001
**Insulin, uIU/mL (SD)**	7.5 (3.9)	8.9 (4.1)	11.1 (5.4)	12.6 (6.4)	< 0.001
**C-peptide, ng/mL (SD)**	1.62 (0.66)	1.83 (0.69)	2.50 (0.86)	2.63 (0.96)	< 0.001
**HOMA-IR (SD)**	1.76 (1.07)	2.14 (1.16)	2.76 (1.44)	3.23 (2.06)	< 0.001
**BUN, mg/dL (SD)**	13.4 (3.4)	13.4 (3.4)	14.3 (3.4)	13.9 (3.4)	< 0.001
**Creatinine, mg/dL (SD)**	0.88 (0.16)	0.78 (0.14)	1.00 (0.13)	0.88 (0.16)	< 0.001
**eGFR, mL/min per 1.73m^2 (SD)**	83.8 (13.0)	85.7 (14.0)	80.6 (11.9)	84.8 (13.7)	< 0.001
**Urinary albumin-to-creatinine ratio, mg/g (SD)**	6.3 (5.3)	7.3 (5.8)	7.0 (6.2)	8.0 (6.3)	< 0.001

BMI body mass index, *BUN* blood urea nitrogen, *CRP* c-reactive protein, *DBP* diastolic blood pressure, *eGFR* estimated glomerular filtration rate, *HDL-C* high-density lipoprotein cholesterol, *HOMA-IR* homeostasis model assessment of insulin resistance, *LDL-C* low-density lipoprotein cholesterol, *SBP* systolic blood pressure, *SD* standard deviation, *SMI* skeletal muscle mass index, *WC* waist circumference

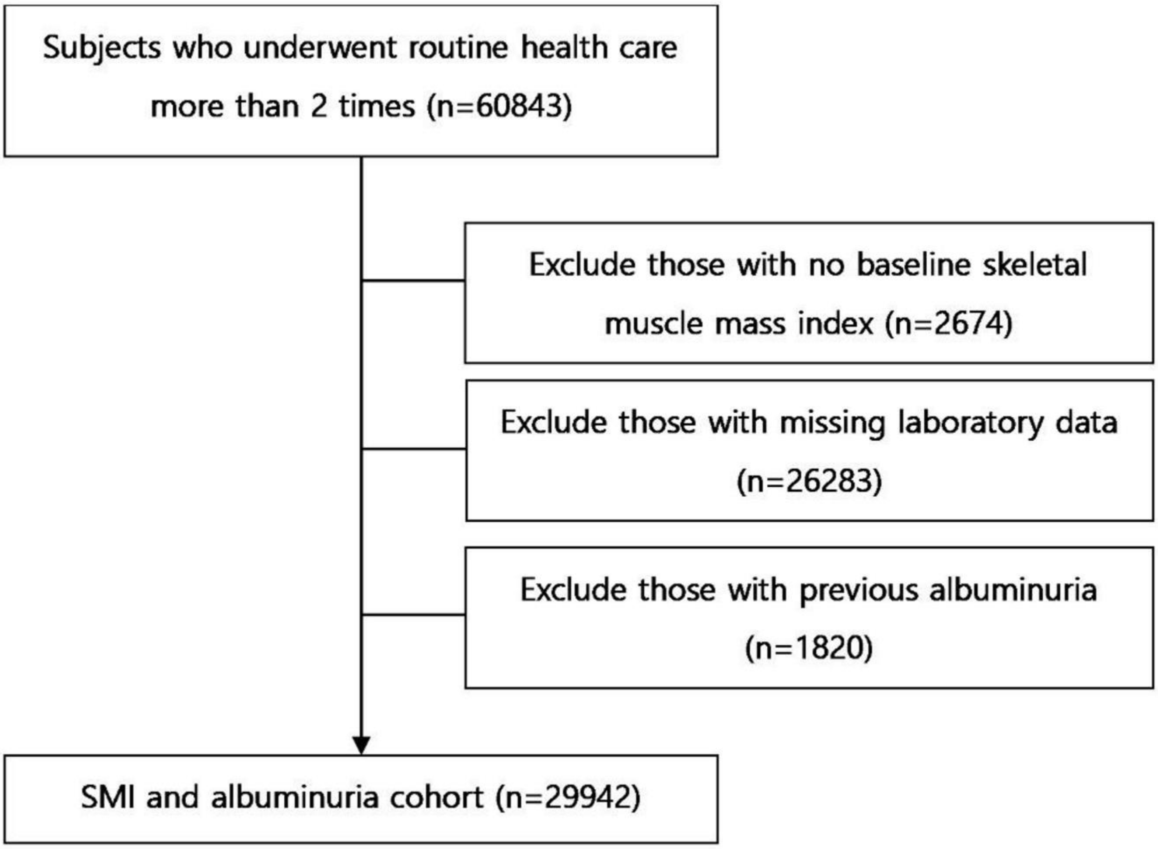

Figure S1 Flowchart of numbers included in the study.
